# Diet affects inflammatory arthritis: a Mendelian randomization study of 30 dietary patterns causally associated with inflammatory arthritis

**DOI:** 10.3389/fnut.2024.1426125

**Published:** 2024-07-17

**Authors:** Haiyang Wang, Qinglin Wu, Pengda Qu, Shiqi Wang, Shiyu Du, Zhaorong Peng, Licheng Tao, Wuxia Wang, Xiaohu Tang

**Affiliations:** ^1^The First Clinical of Medicine College, Yunnan University of Chinese Medicine, Kunming, China; ^2^Department of Rheumatology, Yunnan Provincial Hospital of Traditional Chinese Medicine, Kunming, China

**Keywords:** diet, inflammatory arthritis, Mendelian randomization, causal associations, genome-wide association analysis, inverse variance weighting method, sensitivity analysis

## Abstract

**Background:**

The causal associations between dietary intake and the risk and severity of Inflammatory Arthritis (IA) are currently unknown.

**Objective:**

In this study, we aimed to investigate the causal relationship between nine dietary categories (30 types of diet) and IA using Mendelian randomization (MR).

**Methods:**

We analyzed data from 30 diets and IA in a genome-wide association study (GWAS). Single nucleotide polymorphisms (SNPs) that could influence the results of MR analyses were screened out through the Mendelian Randomization Pleiotropy RESidual Sum and Outlier (MR-PRESSO) test. SNPs were analyzed through two-sample bidirectional MR using inverse variance weighting, MR-Egger regression, and weighted median method. The multiplicity and heterogeneity of SNPs were assessed using MR-Egger intercept term tests and Cochran’s *Q* tests. FDR correction was used to correct the *p*-values.

**Results:**

IVW results showed that Beef intake [Odds ratio (OR) = 2.862; 95% confidence interval (CI), 1.360–6.021, *p* = 0.006, *p_fdr* < 0.05] was positively associated with rheumatoid arthritis(RA); Dried fruit intake (OR = 0.522; 95% CI, 0.349–0.781, *p* = 0.002, *p_fdr* < 0.05), and Iron intake (OR = 0.864; 95%CI, 0.777–0.960, *p* = 0.007, *p_fd*r < 0.05) were negatively associated with RA, all of which were evidence of significance. Fresh fruit intake (OR = 2.528. 95% CI, 1.063–6.011, *p* = 0.036, *p_fdr* > 0.05) was positively associated with psoriatic arthritis (PsA); Cheese intake (OR = 0.579; 95% CI, 0.367–0.914, *p* = 0.019, *p_fdr* > 0.05) was negatively associated with PsA; both were suggestive evidence. Processed meat intake (OR = 0.238; 95% CI, 0.100–0.565, *p* = 0.001, *p_fdr* < 0.05) was negatively associated with reactive arthritis (ReA), a protective factor, and significant evidence. All exposure data passed the heterogeneity check (Cochrane’s *Q* test *p* > 0.05) and no directional pleiotropy was detected. Leave-one-out analyses demonstrated the robustness of the causal relationship in the positive results.

**Conclusion:**

Our study presents genetic evidence supporting a causal relationship between diet and an increased risk of IA. It also identifies a causal relationship between various dietary modalities and different types of IA. These findings have significant implications for the prevention and management of IA through dietary modifications.

## 1 Background

Inflammatory Arthritis (IA) is a chronic inflammatory disease that is characterized by elevated inflammatory factors, pain, joint destruction, and decreased patient function (1, 2). Currently, IA is a significant source of pain and joint disability on a global scale, greatly impacting the well-being of individuals and placing a substantial financial strain (3–5). IA comprises several subtypes, including ankylosing spondylitis (AS), reactive arthritis (ReA), psoriatic arthritis (PsA), and rheumatoid arthritis (RA) (2). Among these, RA has been the most extensively studied. Although the pathogenesis is not yet fully understood, several risk factors have been identified, such as environmental factors, obesity, metabolic levels, and smoking (6–9). Taking action early to tackle these potential risks can enhance the outcome of the illness.

Dietary factors are strongly associated with IA, with vegetarian (10) and Mediterranean diets (11) promoting a decrease in inflammatory markers, while diets high in sugar, saturated fatty acids, and cholesterol increase the number of inflammatory markers (12), and previous studies have reported that dietary patterns may affect joint symptoms (13), and that Inflammatory dietary patterns may lead to an increased risk of RA in the female population, whereas healthier diets may reduce the risk of the disease (14, 15). As the pathogenesis of inflammatory arthritis has been intensively studied, it has been found that abnormalities in iron metabolism play an important role in the disease process. Iron is a key component of many biological processes, but excess iron can exacerbate joint damage by promoting oxidative stress and inflammatory responses (16). Investigating whether dietary modifications are beneficial for patients with IA has positive implications for both patients and physicians. Previous studies have reported that excessive consumption of red meat increases the risk of early-onset RA (17), and dietary fiber supplementation reduces the activity of the disease in patients with AS (18). However, it has been argued that several current cross-sectional studies, with limited sample sizes and confounding factors affecting the results, cannot be relied upon to conclude that there is a correlation between diet and IA (19). The causal associations between other dietary intake and the risk and severity of IA are currently unknown.

To demonstrate whether a causal relationship exists between the observed correlation between diet and IA, MR analyses can be performed (20). MR simulates the process of random group assignment in clinical randomized controlled trials by using randomly distributed single nucleotide polymorphisms (SNPs) in genetic data as instrumental variables (IVs) for group assignment (21–23). Mendel’s second law states that individuals are randomly assigned alleles. Since alleles are fixed at the time of formation of the fertilized egg, the use of the MR method circumvents the effects of reverse causality and confounding environmental factors inherent in the traditional epidemiological RCT approach, making the study conclusions more reliable.

We utilized an MR study design to thoroughly and objectively examine the connection between dietary habits and IA, exploring the potential causal links between thirty different types of diets categorized into nine dietary groups and IA.

## 2 Materials and methods

### 2.1 Study design

**Figure 1:**
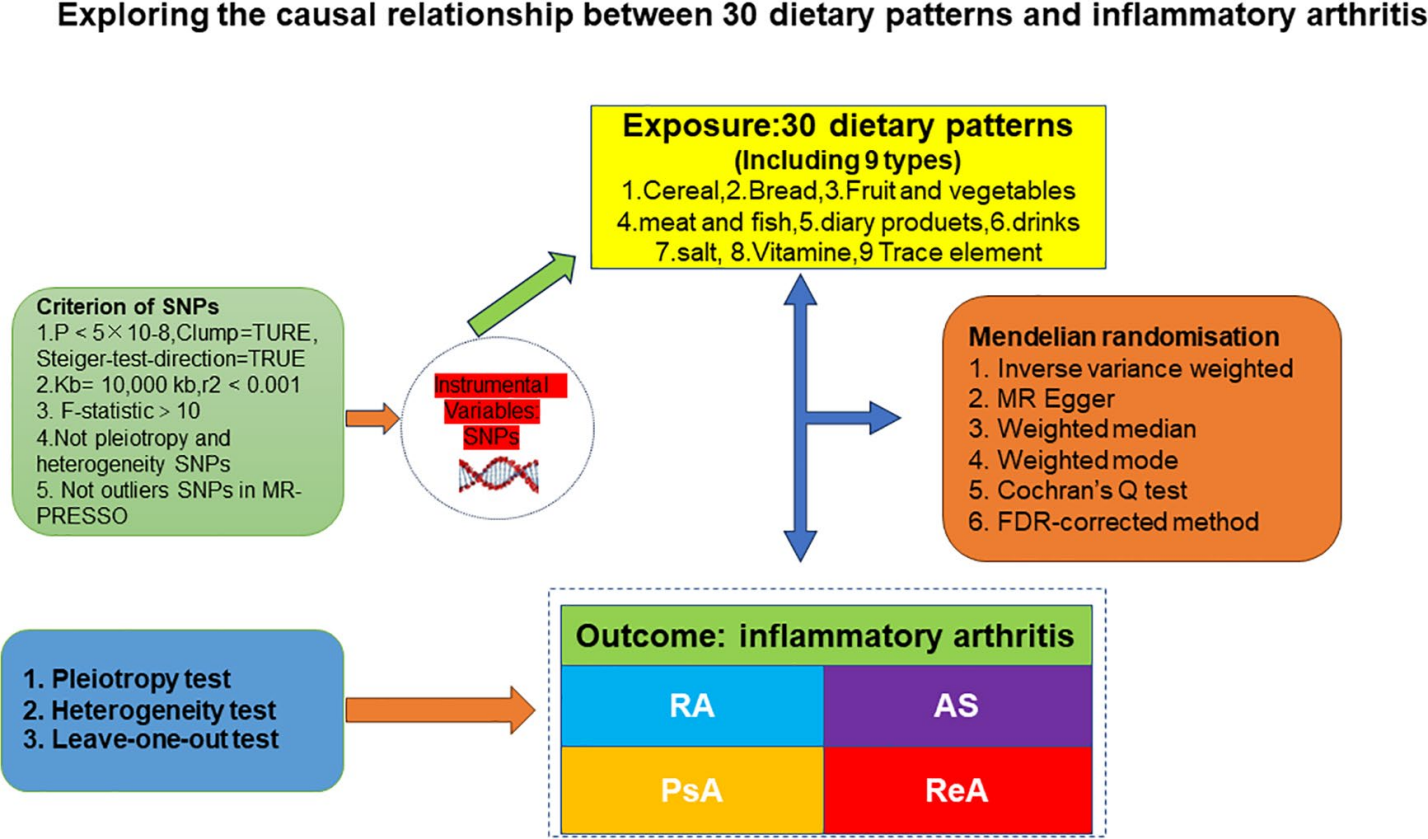
A flowchart of study design. AS, ankylosing spondylitis; ReA, reactive arthritis; PsA, 545 psoriatic arthritis; RA, rheumatoid arthritis; MR-PRESSO, MR Pleiotropy RESidual Sum and 546 Outlier.

We used data from the Genome-Wide Association Study (GWAS) of Diet and IA to perform a comprehensive MR analysis using the TSMR methodology with 30 dietary intakes as exposures and four IAs subtypes as endpoints to clarify the causal relationship between the two. The MR analysis was premised on the following basic assumptions (24): 1. When using SNPs as instrumental variables, it is important that they are strongly associated with the risk factors being studied (correlation hypothesis). Additionally, the genetic variants used should not be associated with potential confounders (independence hypothesis). Finally, it is crucial that genetic variants only influence the risk of an outcome through the associated risk factors and not through any other pathways (exclusionary restriction hypothesis). (See Figure 1 for details).

### 2.2 Data sources

The 30 dietary exposures used in this study were from populations of European descent (data from a web-based questionnaire sent to approximately 330,000 participants by researchers at BioBank UK, including information on diet and food preferences, aged 40–69 years with a roughly even number of men and women recruited between 2006 and 2010 throughout the UK. for more details see: www.ukbiobank.ac.uk/enable-your-research/about-our-data/questionnaire-data). GWAS summary level data for the diet types published by the IEU Open GWAS project. The diet primarily consisted of essential foods, including cereals, bread, various meats (beef, lamb/mutton, pork, oily fish, non-oily fish, poultry, processed meat), vegetables (salad/raw vegetables, cooked vegetables), fresh and dried fruits, cheese, and beverages (alcoholic drinks per week, tea, water, coffee). Additionally, vitamin supplements (A, B, C, D, E) and micronutrients (iron, copper, zinc, magnesium, calcium, selenium) were considered, as well as salt used in food preparation. IA GWAS summary data were extracted from the FinnGen Biobank r10 study updated in December 2023 (which is regularly updated and conducted mainly in the Finnish population, with rigorous inclusion of diagnostic criteria and essentially no overlap with populations with exposure factors). Four main subtypes were included: RA, AS, PsA, and ReA. In order for a discharge diagnosis to be considered appropriate, it must meet the following disease classification criteria (RA: ICD-10: M05, M06, AS: ICD-10:M45, PsA: ICD-10: M07.0*, M07.0*L40.5, M07.1*, M07.1*L40.5, M07.2*, M07.2*L40.5, M07.3*, M07.3*L40.5, ReA: ICD-10:M02, M03). More information on the exposure and outcome datasets (GWAS ID, population, sample size, number of SNPs, etc.) is provided in Supplementary Table S1. As the data used in this study was publicly accessible, anonymized, and de-identified, no ethical review board approval was required.

### 2.3 Selection of instrumental variables

SNPs associated with dietary factors were extracted from the IEU Open GWAS project.^1^ To satisfy the relevance assumption, we screened SNPs that were closely associated with exposure at the genome-wide significance level (*p* < 5 × 10^−8^), had an aggregation window of more than 10,000 kb, and had a low linkage disequilibrium level (*r*^2^ < 0.001) to ensure their independence from each other (25). To meet the independence and exclusivity assumptions, the PhenoScannerV2 database^2^ was searched for these SNPs strongly associated with dietary factors, and those associated with confounders (e.g., smoking behavior and BMI) and outcome variables (IA) were manually excluded. SNPs were screened to ensure that the screened SNPs were unrelated to potential confounders between exposure and outcome and were not directly related to the outcome, but could only be causally related via exposure (26). If there was an SNP with an indirect effect that was associated with confounders and outcome variables (*p* < 0.001), it was excluded from the screened SNPs, i.e., instrumental variables. Finally, the *F* statistic was used to confirm a strong association between the independent variables and exposure. An *F* statistic greater than 10 is generally considered to satisfy this requirement (27). The *F* value was calculated as follows: *F* = (beta/se)^2^ (28) and SNPs with a significant level of correlation with the results (*p* < 5 × 10^−5^) were also excluded from the analysis to satisfy the independence assumption. Finally, the SNPs that simultaneously satisfied all three main hypotheses were screened to be used as instrumental variables for further instrumental variables for MR analysis.

### 2.4 Statistical analysis

The TSMR method was used in this study to estimate the causal effects of several dietary factors on IA. Inverse variance weighted (IVW) analysis was used as the primary outcome, which has strong power to detect causality due to its assumption that the instrument can only affect the outcome through exposure and not through any other alternative pathways (the intercept is restricted to zero) (29). Although known confounding SNPs have been removed as far as possible, there are still many unknown confounders that may bias the results. Therefore, MR-Egger, weighted median, weighted mode and other analytical methods were also used to complement the IVW analysis (30). It should be noted that the MR-Egger method with the weighted median method can provide more robust estimates in a wider range of scenarios, but the effect size will be small (30). Weighted models are more sensitive to the choice of model estimation bandwidth (31). Results are more convincing when all models are consistent.

### 2.5 Sensitivity analysis

The heterogeneity of individual genetic variance estimates was assessed by Cochran’s *Q*-test (32), where *p* > 0.05 for Cochran’s *Q*-test means that there is no heterogeneity among SNPs. Potential horizontal multiplicity was tested by MR-Egger intercept (33), and if *p* > 0.05, it indicates that there is no horizontal multiplicity in the study. Sensitivity analyses were also performed using the leave-one-out method to observe the magnitude of the effect of individual SNPs on causality after the final inclusion of SNPs was eliminated one by one. We also used the MR-PRESSO method to detect outliers. If an outlier appeared, it was immediately removed. After removing the outliers, the MR analysis was performed again.

The study focused on the causal relationship between various dietary factors and IA. The reliability of the results was verified through sensitivity analyses and False Discovery Rate (FDR) correction (*p*-value correction) (34). *p*-values less than 0.05 for both the original *p*-value and the FDR correction were considered indicative findings. The examination was carried out utilizing TwoSampleMR (v0.5.8), MendelianRandomization (v0.8.0), and MRPRESSO package (v1.0) within R software 4.3.1.^3^

## 3 Results

### 3.1 Description of instrumental variables

An investigation was conducted on thirty exposures to explore the correlation between dietary elements and IA. The exposure samples were exclusively from people of European ancestry, with varying sample sizes between 2,603 and 462,630. Outcomes from four IA samples of European ancestry from the FinnGen Biobank were collected, with sample sizes varying between 265,902 and 297,932. There was minimal intersection between the groups exposed and the groups experiencing the outcomes. Supplementary Table S1 contains further details regarding the exposure and outcome. SNPs strongly associated with the four IA species were screened (*p* < 5×10^−8^), and linkage disequilibrium was removed (*r*^2^ = 0.001, kb = 10,000). After exclusion of SNPs with potential associations with confounders or outcome variables by the PhenoScannerV2 database and detection of outliers by the MR-PRESSO method, the following SNPs were excluded: Diet and RA Exclusion SNPs (“rs2279844,” “rs9919429,” “rs1421085,” “rs56094641,” “rs2289292,” “rs4603502”), Diet and AS Exclusion SNPs (“rs7641973,” “rs2263636”), Diet and PsA Exclusion SNPs (“rs746868,” “rs4665972”), Diet and ReA Exclusion SNPs (“rs656817,” “rs4318925”). Finally, 2,638 SNPs were screened that simultaneously met the three major hypotheses, and the largest *F* value was found to be 926.996 and the smallest 29.740 by calculating the *F* statistic. The *F* values of all the SNPs were greater than 10, suggesting that dietary factors and IA—related phenotypes were less susceptible to bias from weak instrumental variables. Further information can be found in Supplementary Table S2.

### 3.2 Results of two-sample MR analysis of dietary factors in four inflammatory joints

### 3.2.1 Diet and RA

**Figure 2:**
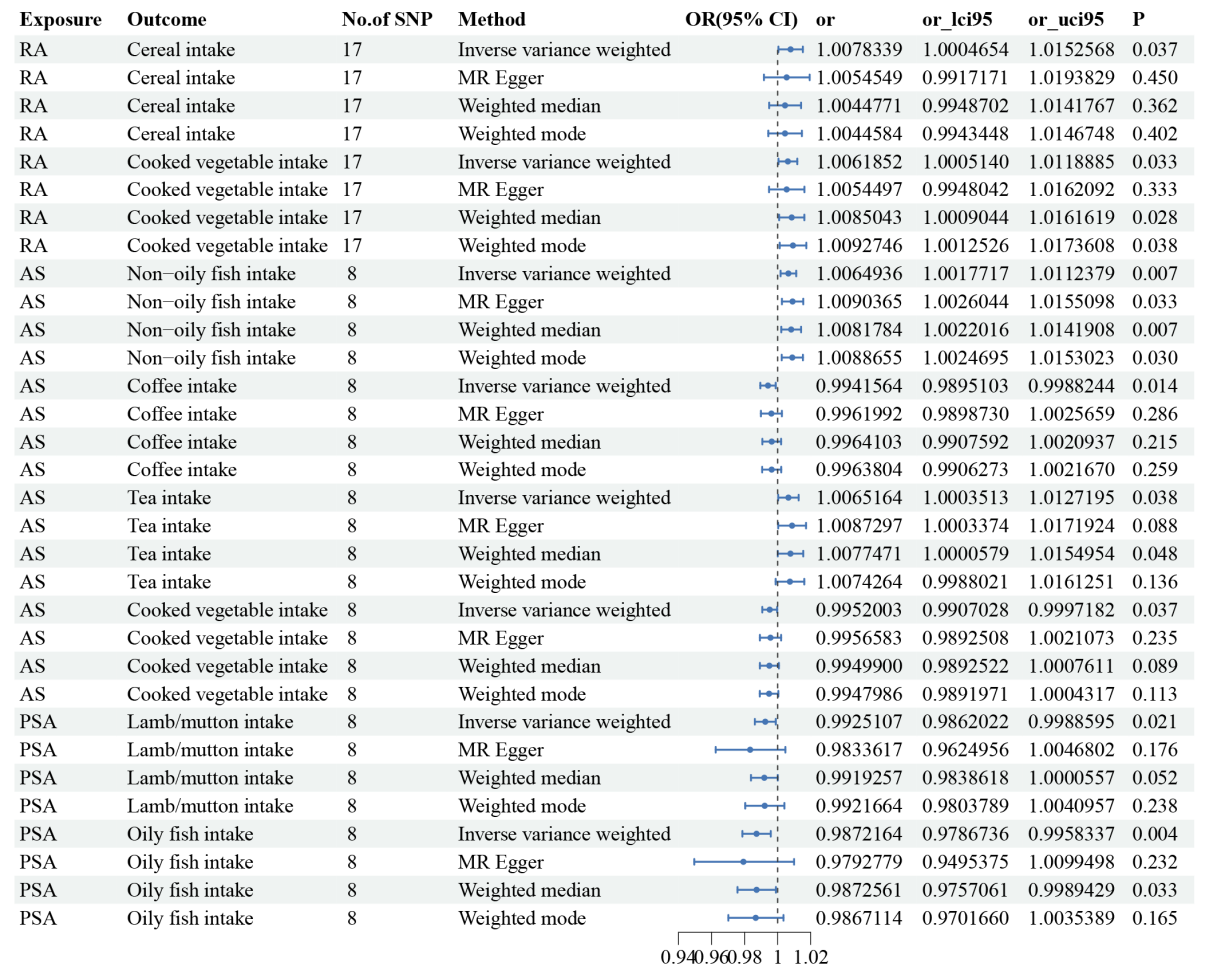
The positive results of TSMR analysis for associations between 30 dietary intake patterns and inflammatory arthritis.

**Figure 3:**
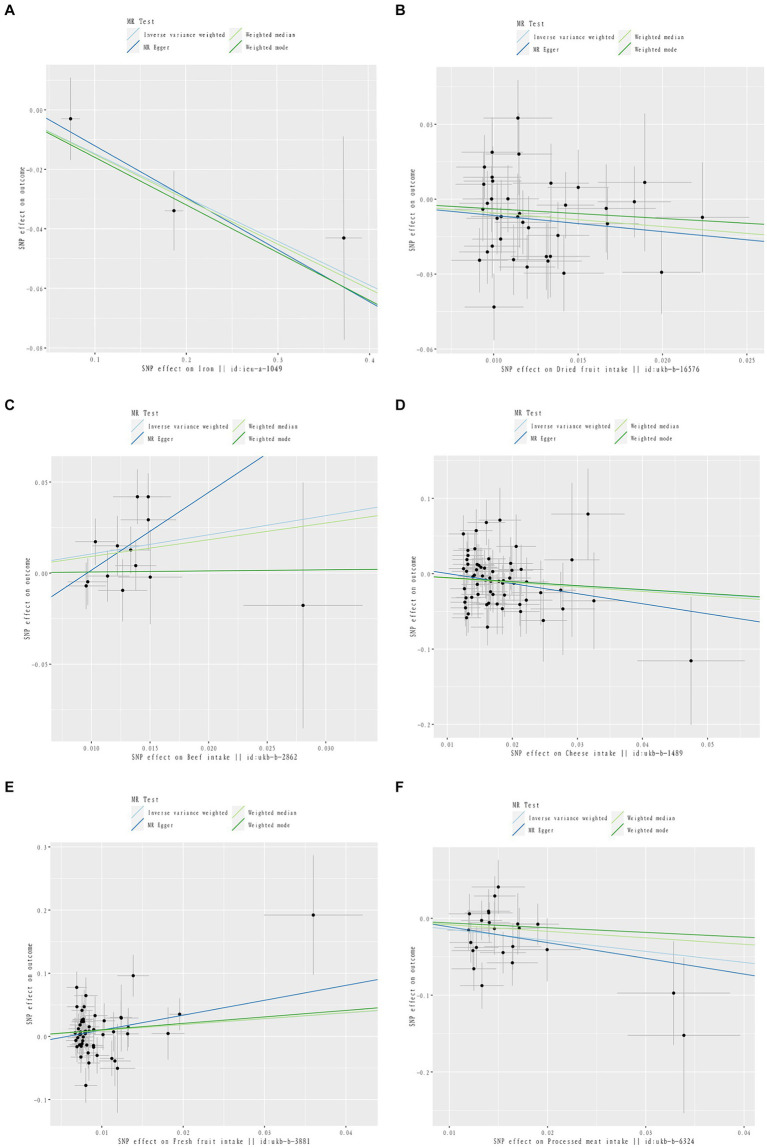
Scatter plot of causal relationship between dietary intake and different phenotypes of inflammatory arthritis. The causal effects of iron intake on RA in different MR methods **(A)**. The causal effects of dried fruit intake on RA in different MR methods **(B)**. The causal effects of Beef intake on RA in different MR methods **(C)**. The causal effects of Cheese intake on PsA in different MR methods **(D)**. The causal effects of Fresh fruit intake on PsA in different MR methods **(E)**. The causal effects of processed meat intake on ReA in different MR methods **(F)**.

**Figure 4:**
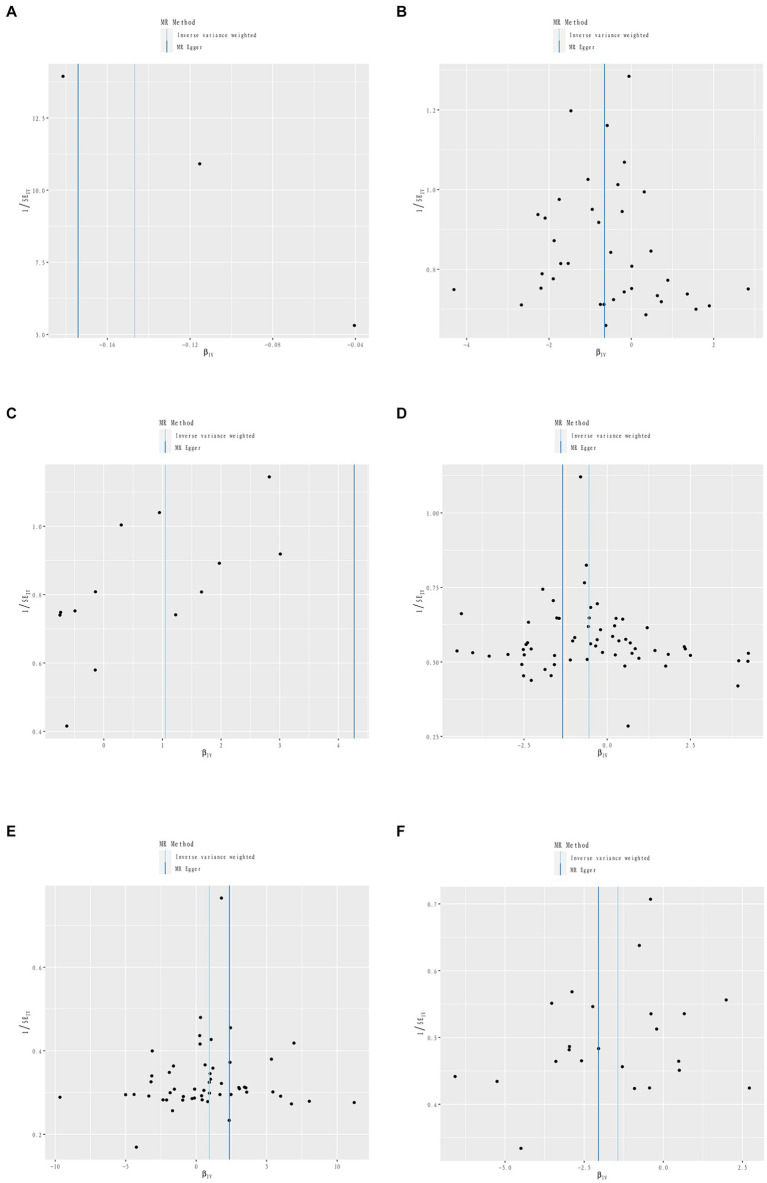
Sensitivity analysis funnel plot. Funnel plot of sensitivity analysis between Iron intake and RA **(A)**. Funnel plot of sensitivity analysis between Dried fruit intake and RA **(B)**. Funnel plot of sensitivity analysis between Beef intake and RA **(C)**. Funnel plot of sensitivity analysis between Cheese intake and PsA **(D)**. Funnel plot of sensitivity analysis between Fresh fruit intake and PsA **(E)**. Funnel plot of sensitivity analysis between Processed meat intake and ReA **(F)**.

**Figure 5:**
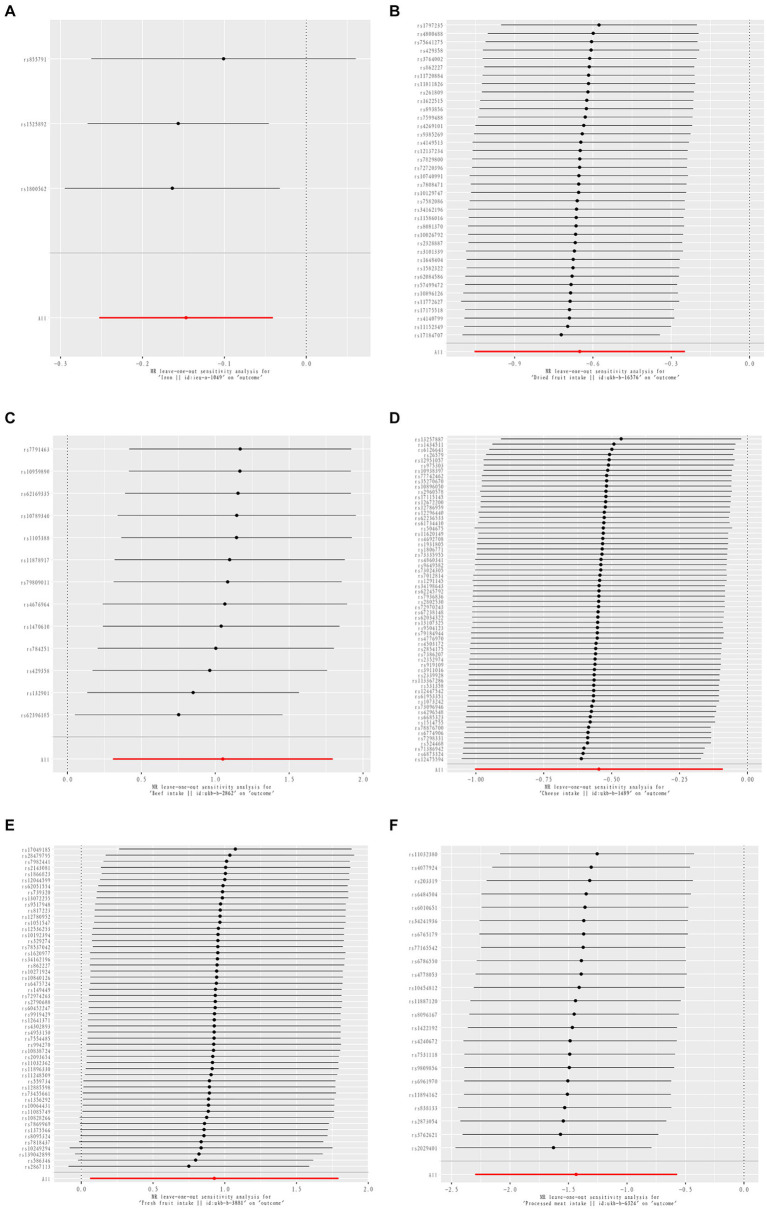
MR leave-one-out sensitive analysis for Iron intake on RA **(A)**. MR leave-one-out sensitive analysis for Dried fruit intake on RA **(B)**. MR leave-one-out sensitive analysis for Beef intake on RA **(C)**. MR leave-one-out sensitive analysis for Cheese intake on PsA **(D)**. MR leave-one-out sensitive analysis for Fresh fruit intake on PsA **(E)**. MR leave-one-out sensitive analysis for processed meat intake on ReA **(F)**.

Three dietary factors and RA were statistically significant in MR analysis, with Beef intake as a risk factor, Dried fruit intake, and Iron intake as a protective factors. IVW results showed that Beef intake [Odds ratio (OR) = 2.862; 95% confidence interval (CI), 1.360–6.021, *p* = 0.006, *p_fdr* < 0.05] was positively associated with RA; Dried fruit intake (OR = 0.522; 95% CI, 0.349–0.781, *p* = 0.002, *p_fdr* < 0.05), Iron intake (OR = 0.864; 95% CI. 0.777–0.960, *p* = 0.007, *p_fdr* < 0.05) were negatively associated with RA, all of which were significant evidence. The *p*-value for heterogeneity of the above dietary factors were all >0.05, and MR Egger found no evidence of horizontal pleiotropy. Re-assessment of pleiotropy using MRPRESSO also revealed no outliers and the *P-fdr* adjusted *p*-value of <0.05 also indicated that the IVW results were reliable (Refer to Table 1 and Figures 2–5).

**Table 1:** Anisotropy and heterogeneity test results of two sample MR analysis with positive results.

Exposure	Outcome	Ethnic	Pleiotropy_test (MR-Egger)	Heterogeneity_test						MR-PRESSO (Global test)	
			*p*_value	Method	*Q* statistic	*p*_value	Method	*Q* statistic	*p*_value	RSSobs	*p*_value
Iron || id:ieu-a-1049	RA	European	0.826	MR-Egger	0.591	0.442	IVW	0.670	0.715	NA	NA
Dried fruit intake || id:ukb-b-16576	RA	European	1.000	MR-Egger	44.296	0.161	IVW	44.296	0.191	46.421	0.207
Beef intake || id:ukb-b-2862	RA	European	0.141	MR-Egger	12.769	0.309	IVW	15.684	0.206	19.089	0.210
Cheese intake || id:ukb-b-1489	PsA	European	0.425	MR-Egger	68.610	0.209	IVW	69.347	0.217	71.374	0.231
Fresh fruit intake || id:ukb-b-3881	PsA	European	0.327	MR-Egger	60.817	0.163	IVW	61.983	0.162	64.204	0.167
Processed meat intake || id:ukb-b-6324	ReA	European	0.787	MR-Egger	24.574	0.266	IVW	24.662	0.313	26.809	0.337

#### 3.2.2 Diet and AS

Non-oily fish intake and AS were statistically significant in MR analysis, Non-oily fish intake (OR = 4.274; 95% CI, 1.025–17.822, *p* = 0.046, *p_fdr* > 0.05). As the *p-fdr* adjusted *p*-value was >0.05, it can only be considered as suggestive evidence. However, subsequent reverse causality validation revealed a reverse causality between non-oily fish intake (OR = 1.006; 95% CI, 1.002–1.011, *p* = 0.007) and AS (Refer to Supplementary Figure S1). Consequently, the analysis was adjusted to exclude this variable. Ultimately, the results indicated that there is no causal relationship between dietary factors and AS.

#### 3.2.3 Diet and PsA

The 2 dietary factors were statistically associated with PsA in MR analysis, with fresh fruit intake as a risk factor and Cheese intake as a protective factor. IVW results showed that Fresh fruit intake (OR = 2.528; 95% CI, 1.063–6.011, *p* = 0.036, *p_fdr* > 0.05) was positively associated with PsA; Cheese intake (OR = 0.579; 95% CI, 0.367–0.914, *p* = 0.019, *p_fdr* > 0.05) was negatively associated with PsA, both of which were suggestive evidence. All these results passed the heterogeneity and multiple validity tests. (Refer to Table 1 and Figures 2–5).

#### 3.2.4 Diet and ReA

1 dietary factors were statistically significant with ReA in MR analysis, and IVW results showed that processed meat intake (OR = 0.238; 95% CI, 0.100–0.565, *p* = 0.001, *p_fdr* < 0.05) was negatively correlated with ReA as a protective factor, and all of the above results passed the heterogeneity and multivalence tests, and the *p-fdr* adjusted *p*-value <0.05 also indicates that the IVW results are reliable. (Refer to Table 1 and Figures 2–5).

### 3.3 Inverse Mendelian randomization study

To avoid reverse causality influencing the above findings, we performed reverse MR analyses with IA as the exposure and 30 dietary factors as the outcome, and found that, except for reverse causality between AS and non-oily fish intake which was excluded, no reverse causality influence was found for other positive results, indicating that the results of the MR analyses were very robust (see Supplementary Figure S1).

All exposure data passed the heterogeneity check (Cochrane’s *Q* test *p* > 0.05) and no directional pleiotropy was found. Leave-one-out analyses showed very robust causality for the positive results. The MR-PRESSO analyses yielded results consistent with those of the IVW model, indicating the reliability of the study results.

The full findings of the positive and reverse MR studies, as well as the results of the heterogeneity test and the pleiotropy test, are shown in Supplementary Tables S3–S26.

## 4 Discussion

In this study, MR was used to investigate the potential causal associations between 30 dietary factors and IA, and it was found that Beef intake, Dried fruit intake, Iron intake, Fresh fruit intake, Cheese intake, and Processed meat intake were strongly associated with IA. The *p*-values and *p_fdr* values for the associations between Beef intake and RA, Dried fruit intake and RA, Iron intake and RA, and Processed meat intake and ReA were all less than 0.05, indicating more reliable results. In addition, our study found no causal relationship between the rest of the dietary factors and inflammatory joints.

Previous MR studies have shown that the onset of several chronic diseases, such as cardiovascular-metabolic diseases, psychiatric disorders, oesophageal disorders, tumors and asthma, is strongly associated with dietary factors (35–39), and the progression of these chronic diseases may be related to a long-term chronic inflammatory state influenced by dietary factors (10, 40). Unhealthy diets, such as those high in sugar, salt, trans fats, and ultra-processed foods, can lead to imbalances in the gut microbiota, increased oxidative stress, and activation of inflammatory genes. In addition, vitamin and mineral deficiencies and insufficient intake of omega-3 fatty acids have also been linked to inflammation. Healthy eating habits, such as increased intake of fruits, vegetables, and fiber-rich foods, may help to reduce inflammation (41) and may also assist in regulating gut microbiota diversity and stability to prevent disease (42).

IA, a major disease characterized by a long-term chronic inflammatory state, has become increasingly prevalent in recent years, and the identification of modifiable risk factors (e.g., dietary factors) is an achievable way to halt the onset and progression of this type of disease. To overcome the problem of the low inclusion of dietary factors and the homogeneity of the diseases studied in previous studies, we conducted this comprehensive study to assess the causal relationship between several dietary factors (cereals, bread, vegetables and fruits, meat and fish, beverages, dairy products, salt, vitamins, minerals) and IA.

Cereals may be associated with IA, and an observational cohort study of Swedish women found that women without RA consumed more whole-grain bread compared with women with RA (43). The mechanism of action may be related to the ability of whole-grain breads to improve inflammation by increasing total plasma polyphenol and antioxidant status (44). Our study did not find an association between the two at a genetic level, and the relationship could be further clarified in the future by conducting causal association studies with data on different types of cereals and IA.

Prior research has shown that fruits and vegetables are crucial for maintaining good health due to their nutrient content and anti-inflammatory properties (45). Consuming fresh fruits and vegetables is beneficial in RA and may be related to the fact that macronutrients and micronutrients modulate the pathogenesis of RA by regulating the migration and activity of neutrophils (46). However, our study did not find a causal association between the two, and even in the PsA causal association study, we found Fresh fruit intake as a risk factor. The possible reasons for the above discrepancies are as follows. Although the statistical data analysis was performed at 0.05 level of significance for two-sided analysis, it was not validated by FDR correction method (*p* > 0.05), suggesting that there may be false positive results. 2. Although it has been adjusted for possible confounders, observational studies may still be affected by other confounders. 3. One study on the relationship between diet and PsA found that a vegetable- and fruit-based Mediterranean diet or supplementation was associated with the disease, but there is a lack of high-quality evidence to draw the above conclusions (47). Another clinical study also concluded only that higher levels of disease activity, as measured by the Psoriatic Arthritis Disease Activity Index, were associated with low adherence to the Mediterranean diet, with no clear evidence that high adherence to the Mediterranean diet reduces disease activity in PsA (48), suggesting that more large-scale randomized trials and more subtle MR studies are needed in the future to confirm the relationship.

There is a paucity of research examining the impact of dried fruits on IA. Our study offers compelling evidence of a causal relationship between dried fruits and RA. Prior studies have identified a range of bioactive compounds, dietary fibers, and vitamins in dried fruits, which act as natural antioxidants and may play a protective role by reducing oxidative stress and cartilage cell damage (49). This highlights the potential importance of dried fruits in the development of nutritional interventions for patients with RA. Clinicians should consider the potential benefits of incorporating dried fruits into nutritional interventions for RA patients.

Meat intake, especially red meat, increases the risk of IA (17). The present study also found that beef intake increases the risk of RA, which has been confirmed in previous MR studies (50). It is puzzling that in a study of the causality of dietary factors in ReA, we found that intake of processed meat was a protective factor against the disease, which is generally considered to increase the risk of developing chronic disease, but previous studies have focused more on the association of processed meat with the risk of tumours, diabetes mellitus, and cardiovascular diseases (51). There are no observational case reports of the association of processed meat with ReA, and further studies are needed on the causality of processed meat and IA.

There is some controversy regarding the relationship between alcohol consumption and IA, and our study did not find a causal relationship between alcohol intake and IA. Previous studies have found that resveratrol (an important component of red wine) has significant anti-inflammatory and immunomodulatory properties and can intervene in joint inflammation and improve symptoms through a variety of pathways (52, 53). Dietary polyphenols down-regulate inflammatory cytokines, enhance antioxidant defenses, and inhibit inflammatory pathways (54). However, Niemelä Onni et al. (55). found that even at fairly low levels of alcohol consumption, all forms of alcohol consumption were associated with increased liver function, lipid status, and inflammatory markers, suggesting that patients with IA need to be aware of the need to reduce the frequency of alcohol consumption, the amount of alcohol consumed and, if necessary, the type of alcohol that is more beneficial to health.

Many studies have shown that dairy products are beneficial for bone health because of their richness in protein minerals and probiotics (56, 57). Our study also found that cheese intake was a protective factor in PsA, which may be related to the fact that cheese is beneficial to the musculoskeletal system and can help increase bone mass (58). However, there are fewer relevant clinical studies, and it was only possible to retrieve a single article on the association between dairy consumption and RA A cross-sectional study on the correlation (59) suggests the need to further increase the number of dairy products and other IA cases and experimental studies in future clinical practice.

Trace elements play a role in IA as effectors of the immune system, inflammation, and metabolism, and previous studies have found that zinc, an important human protein cofactor and signaling ion, affects many of the pathways associated with arthritic disease (60). Low magnesium has been associated with the presence of osteoporosis, and supplemental intake of magnesium may improve bone density and reduce the risk of fracture (61). We have also examined the relationship between iron, copper, zinc, magnesium, and calcium, selenium, six micronutrients, to IA and found that iron intake was a protective factor in RA. Iron deficiency anemia is not uncommon in patients with rheumatoid arthritis, and iron supplementation appears to be beneficial in patients with RA. However, previous studies have found iron deficiency in the blood and abnormal accumulation of iron in the synovial membrane and synovial fluid in RA patients, which may suggest redistribution of iron in the body. Excessive accumulation of iron in synovial cells and lipid peroxidation can lead to structural abnormalities in the mitochondria, causing cellular dysfunction and aggravation of RA, so iron supplementation in patients with RA needs to be taken with great caution (16). A US cohort study of dietary factors and RA risk covering 82,063 women did not find an association between iron levels and RA (62). It cannot be ruled out that the positive results for iron intake were affected by confounding factors such as levels of inflammation in RA (63), inflammatory anemia (64), and other factors affecting the distribution of iron in the body.

Although many studies have found an association between other factors such as vitamin supplementation, appropriate coffee citation, and improvement in IA disease activity, the fact that our study did not find a causal association cannot be excluded from being related to the insufficient sample size for the inclusion of several exposure factors of interest in the study, suggesting that the sample size should be continued to be expanded in the future to obtain more stable results.

This study is the first to investigate the causal relationship between multiple dietary intakes and IA using MR analysis. The approach used in this study avoids confounding and reverse causality. Previous studies on diet and IA have mainly focused on the effects of a single dietary factor. Our study enabled a thorough evaluation of the correlation between various dietary factors and IA. However, it is important to note that the MR analysis was conducted solely on a European population, which may limit the generalizability of our findings to other ethnic groups. It was not possible to investigate the effect of dietary factors on IA in various population subgroups, such as age, gender, and place of origin. Furthermore, there may have been some overlap in the cohorts used to determine exposures and outcomes. Additionally, the *F*-statistic of the IV was sufficient to prevent bias due to weak instrumental variables. However, caution is needed when interpreting the results of our study due to the lack of research on the effects of a combined dietary approach on IA.

## 5 Conclusion

Our study presents genetic evidence supporting a causal relationship between diet and an increased risk of IA. It also identifies causal relationships between several dietary modalities and different types of IA. These findings have significant implications for the prevention and management of IA through dietary modification.

## Data availability statement

The original contributions presented in the study are included in the article/Supplementary material, further inquiries can be directed to the corresponding author.

## Author contributions

HW: Conceptualization, Writing – original draft, Writing – review & editing, Methodology. QW: Conceptualization, Writing – review & editing. PQ: Methodology, Writing – original draft. SW: Formal analysis, Validation, Writing – original draft. SD: Methodology, Validation, Writing – review & editing. ZP: Methodology, Validation, Writing – review & editing. LT: Writing – original draft. WW: Writing – review & editing. XT: Conceptualization, Supervision, Writing – review & editing.

## Footnotes

^1^ https://gwas.mrcieu.ac.uk/

^2^ https://www.phenoscannermedschl.cam.ac.uk/

^3^ https://www.R-project.org

## References

[1] Rausch Osthoff AK Niedermann K Braun J Adams J Brodin N Dagfinrud H. 2018 Eular recommendations for physical activity in people with inflammatory arthritis and osteoarthritis. Ann Rheum Dis. (2018) 77:1251–60. doi: 10.1136/annrheumdis-2018-213585 29997112

[2] Ramiro S Radner H van der Heijde D van Tubergen A Buchbinder R Aletaha D. Combination therapy for pain Management in Inflammatory Arthritis (rheumatoid arthritis, ankylosing spondylitis, psoriatic arthritis, other Spondyloarthritis). Cochrane Database Syst Rev. (2011) 10:Cd008886. doi: 10.1002/14651858.CD008886.pub2 PMC12416524 21975788

[3] Bergman MJ. Social and economic impact of inflammatory arthritis. Postgrad Med. (2006) Spec No:5-11. 17960689

[4] Dinas PC on behalf of the students of module 5104 (Introduction to Systematic Reviews) Moe RH Boström C Kosti RI Kitas GD. Combined effects of diet and physical activity on inflammatory joint disease: a systematic review and Meta-analysis. Healthcare. (2023) 11:1427. doi: 10.3390/healthcare11101427, PMID: 37239713 PMC10218217

[5] Wu X. Innate lymphocytes in inflammatory arthritis. Front Immunol. (2020) 11:565275. doi: 10.3389/fimmu.2020.565275, PMID: 33072104 PMC7544949

[6] Lahiri M Luben RN Morgan C Bunn DK Marshall T Lunt M. Using lifestyle factors to identify individuals at higher risk of inflammatory polyarthritis (results from the European prospective investigation of Cancer-Norfolk and the Norfolk arthritis register--the Epic-2-Noar study). Ann Rheum Dis. (2014) 73:219–26. doi: 10.1136/annrheumdis-2012-202481 23505230 PMC3888611

[7] Lu B Hiraki LT Sparks JA Malspeis S Chen CY Awosogba JA. Being overweight or obese and risk of developing rheumatoid arthritis among women: a prospective cohort study. Ann Rheum Dis. (2014) 73:1914–22. doi: 10.1136/annrheumdis-2014-205459 25057178 PMC4207219

[8] Solmaz D Eder L Aydin SZ. Update on the epidemiology, risk factors, and disease outcomes of psoriatic arthritis. Best Pract Res Clin Rheumatol. (2018) 32:295–311. doi: 10.1016/j.berh.2018.09.006 30527433

[9] Yoshida K Wang J Malspeis S Marchand N Lu B Prisco LC. Passive smoking throughout the life course and the risk of incident rheumatoid arthritis in adulthood among women. Arthritis Rheumatol. (2021) 73:2219–28. doi: 10.1002/art.41939 34406709 PMC8976916

[10] Koelman L Egea Rodrigues C Aleksandrova K. Effects of dietary patterns on biomarkers of inflammation and immune responses: a systematic review and meta-analysis of randomized controlled trials. Adv Nutr. (2022) 13:101–15. doi: 10.1093/advances/nmab086 34607347 PMC8803482

[11] Craddock JC Neale EP Peoples GE Probst YC. Vegetarian-based dietary patterns and their relation with inflammatory and immune biomarkers: a systematic review and meta-analysis. Adv Nutr. (2019) 10:433–51. doi: 10.1093/advances/nmy103 30947338 PMC6520040

[12] Christ A Lauterbach M Latz E. Western diet and the immune system: an inflammatory connection. Immunity. (2019) 51:794–811. doi: 10.1016/j.immuni.2019.09.020 31747581

[13] Tedeschi SK Frits M Cui J Zhang ZZ Mahmoud T Iannaccone C. Diet and rheumatoid arthritis symptoms: survey results from a rheumatoid arthritis registry. Arthritis Care Res. (2017) 69:1920–5. doi: 10.1002/acr.23225 PMC5563270 28217907

[14] Hu Y Sparks JA Malspeis S Costenbader KH Hu FB Karlson EW. Long-term dietary quality and risk of developing rheumatoid arthritis in women. Ann Rheum Dis. (2017) 76:1357–64. doi: 10.1136/annrheumdis-2016-210431 28137914 PMC5556680

[15] Sparks JA Barbhaiya M Tedeschi SK Leatherwood CL Tabung FK Speyer CB. Inflammatory dietary pattern and risk of developing rheumatoid arthritis in women. Clin Rheumatol. (2019) 38:243–50. doi: 10.1007/s10067-018-4261-5 30109509 PMC6344305

[16] Chang S Tang M Zhang B Xiang D Li F. Ferroptosis in inflammatory arthritis: a promising future. Front Immunol. (2022) 13:955069. doi: 10.3389/fimmu.2022.955069 35958605 PMC9361863

[17] Jin J Li J Gan Y Liu J Zhao X Chen J. Red meat intake is associated with early onset of rheumatoid arthritis: a cross-sectional study. Sci Rep. (2021) 11:5681. doi: 10.1038/s41598-021-85035-6 33707573 PMC7952581

[18] Song C Wang L Ji X Wang Y Hu L Liu X. Dietary Fiber intake influences changes in ankylosing spondylitis disease status. J Clin Med. (2023) 12:41621. doi: 10.3390/jcm12041621 PMC9960917 36836155

[19] Maharaj AB Eder L Ogdie A. The impact of dietary interventions in psoriatic arthritis. Curr Opin Rheumatol. (2023) 35:414–22. doi: 10.1097/bor.0000000000000949 37339523

[20] Evangelou E Warren HR Mosen-Ansorena D Mifsud B Pazoki R Gao H. Genetic analysis of over 1 million people identifies 535 new loci associated with blood pressure traits. Nat Genet. (2018) 50:1412–25. doi: 10.1038/s41588-018-0205-x 30224653 PMC6284793

[21] Smith GD Ebrahim S. Mendelian randomization: can genetic epidemiology contribute to understanding environmental determinants of disease? Int J Epidemiol. (2003) 32:1–22. doi: 10.1093/ije/dyg070 12689998

[22] Didelez V Sheehan N. Mendelian randomization as an instrumental variable approach to causal inference. Stat Methods Med Res. (2007) 16:309–30. doi: 10.1177/0962280206077743 17715159

[23] Davey Smith G Hemani G. Mendelian randomization: genetic anchors for causal inference in epidemiological studies. Hum Mol Genet. (2014) 23:R89–98. doi: 10.1093/hmg/ddu328 25064373 PMC4170722

[24] Evans DM Davey SG. Mendelian randomization: new applications in the coming age of hypothesis-free causality. Annu Rev Genomics Hum Genet. (2015) 16:327–50. doi: 10.1146/annurev-genom-090314-050016 25939054

[25] Abecasis GR Altshuler D Auton A Brooks LD Durbin RM Gibbs RA. A map of human genome variation from population-scale sequencing. Nature. (2010) 467:1061–73. doi: 10.1038/nature09534 20981092 PMC3042601

[26] Kamat MA Blackshaw JA Young R Surendran P Burgess S Danesh J. Phenoscanner V2: an expanded tool for searching human genotype-phenotype associations. Bioinformatics. (2019) 35:4851–3. doi: 10.1093/bioinformatics/btz469 31233103 PMC6853652

[27] Burgess S Thompson SG. Avoiding Bias from weak instruments in Mendelian randomization studies. Int J Epidemiol. (2011) 40:755–64. doi: 10.1093/ije/dyr036 21414999

[28] Bowden J Del Greco MF Minelli C Zhao Q Lawlor DA Sheehan NA. Improving the accuracy of two-sample summary-data Mendelian randomization: moving beyond the Nome assumption. Int J Epidemiol. (2019) 48:728–42. doi: 10.1093/ije/dyy258 30561657 PMC6659376

[29] Burgess S Butterworth A Thompson SG. Mendelian randomization analysis with multiple genetic variants using summarized data. Genet Epidemiol. (2013) 37:658–65. doi: 10.1002/gepi.21758 24114802 PMC4377079

[30] Verbanck M Chen CY Neale B Do R. Detection of widespread horizontal pleiotropy in causal relationships inferred from Mendelian randomization between complex traits and diseases. Nat Genet. (2018) 50:693–8. doi: 10.1038/s41588-018-0099-7 29686387 PMC6083837

[31] Hartwig FP Davey Smith G Bowden J. Robust inference in summary data Mendelian randomization via the zero modal pleiotropy assumption. Int J Epidemiol. (2017) 46:1985–98. doi: 10.1093/ije/dyx102 29040600 PMC5837715

[32] Bowden J Del Greco MF Minelli C Davey Smith G Sheehan N Thompson J. A framework for the investigation of pleiotropy in two-sample summary data Mendelian randomization. Stat Med. (2017) 36:1783–802. doi: 10.1002/sim.7221 28114746 PMC5434863

[33] Burgess S Thompson SG. Interpreting findings from Mendelian randomization using the Mr-egger method. Eur J Epidemiol. (2017) 32:377–89. doi: 10.1007/s10654-017-0255-x 28527048 PMC5506233

[34] Benjamini Y Hochberg Y. Controlling the false discovery rate: a practical and powerful approach to multiple testing. J R Stat Soc B. (1995) 57:289–300. doi: 10.1111/j.2517-6161.1995.tb02031.x

[35] Niu YY Aierken A Feng L. Unraveling the link between dietary factors and cardiovascular metabolic diseases: insights from a two-sample Mendelian randomization investigation. Heart Lung. (2024) 63:72–7. doi: 10.1016/j.hrtlng.2023.09.012 37826923

[36] Zhao H Han X Zhang X Li L Li Y Wang W. Dissecting causal associations of diet-derived circulating antioxidants with six major mental disorders: a Mendelian randomization study. Antioxidants. (2023) 12:e162. doi: 10.3390/antiox12010162 PMC9855039 36671024

[37] Zou M Liang Q Zhang W Zhu Y Xu Y. Causal association between dietary factors and esophageal diseases: a Mendelian randomization study. PLoS One. (2023) 18:e0292113. doi: 10.1371/journal.pone.0292113 38019753 PMC10686502

[38] Dong H Kong X Wang X Liu Q Fang Y Wang J. The causal effect of dietary composition on the risk of breast Cancer: a Mendelian randomization study. Nutrients. (2023) 15:112586. doi: 10.3390/nu15112586 PMC10255061 37299548

[39] Yang W Yang Y He L Zhang M Sun S Wang F. Dietary factors and risk for asthma: a Mendelian randomization analysis. Front Immunol. (2023) 14:1126457. doi: 10.3389/fimmu.2023.1126457 36911739 PMC9992976

[40] Medzhitov R. Origin and physiological roles of inflammation. Nature. (2008) 454:428–35. doi: 10.1038/nature07201 18650913

[41] Furman D Campisi J Verdin E Carrera-Bastos P Targ S Franceschi C. Chronic inflammation in the etiology of disease across the life span. Nat Med. (2019) 25:1822–32. doi: 10.1038/s41591-019-0675-0 31806905 PMC7147972

[42] Rinninella E Cintoni M Raoul P Lopetuso LR Scaldaferri F Pulcini G. Food components and dietary habits: keys for a healthy gut microbiota composition. Nutrients. (2019) 11:2393. doi: 10.3390/nu11102393 31591348 PMC6835969

[43] Lourdudoss C Arnaud L Wolk A van Vollenhoven RF Di Giuseppe D. Long-term dietary changes after diagnosis of rheumatoid arthritis in Swedish women: data from a population-based cohort. Int J Rheumatol. (2018) 2018:9152480. doi: 10.1155/2018/9152480 29991946 PMC6016163

[44] Hajira B Khan I. Effect of Sorghum and barley-containing bread on plasma Total polyphenols, antioxidant status and inflammation in healthy subjects. J Food Sci Technol. (2022) 59:4935–44. doi: 10.1007/s13197-022-05582-2 36276540 PMC9579251

[45] Wallace TC Bailey RL Blumberg JB Burton-Freeman B Chen CO Crowe-White KM. Fruits, vegetables, and health: a comprehensive narrative, umbrella review of the science and recommendations for enhanced public policy to improve intake. Crit Rev Food Sci Nutr. (2020) 60:2174–211. doi: 10.1080/10408398.2019.1632258 31267783

[46] Shao YR Xu DY Lin J. Nutrients and rheumatoid arthritis: from the perspective of neutrophils. Front Immunol. (2023) 14:1113607. doi: 10.3389/fimmu.2023.1113607 36923418 PMC10008948

[47] Katsimbri P Korakas E Kountouri A Ikonomidis I Tsougos E Vlachos D. The effect of antioxidant and anti-inflammatory capacity of diet on psoriasis and psoriatic arthritis phenotype: nutrition as therapeutic tool? Antioxidants. (2021) 10:20157. doi: 10.3390/antiox10020157 PMC7912156 33499118

[48] Caso F Navarini L Carubbi F Picchianti-Diamanti A Chimenti MS Tasso M. Mediterranean diet and psoriatic arthritis activity: a multicenter cross-sectional study. Rheumatol Int. (2020) 40:951–8. doi: 10.1007/s00296-019-04458-7 31605152

[49] Alasalvar C Chang SK Kris-Etherton PM Sullivan VK Petersen KS Guasch-Ferré M. Dried fruits: bioactives, effects on gut microbiota, and possible health benefits-an update. Nutrients. (2023) 15:71611. doi: 10.3390/nu15071611 PMC10097306 37049451

[50] Chen W Liu K Huang L Mao Y Wen C Ye D. Beef intake and risk of rheumatoid arthritis: insights from a cross-sectional study and two-sample Mendelian randomization. Front Nutr. (2022) 9:923472. doi: 10.3389/fnut.2022.923472 36147307 PMC9486088

[51] Grosso G La Vignera S Condorelli RA Godos J Marventano S Tieri M. Total, red and processed meat consumption and human health: an umbrella review of observational studies. Int J Food Sci Nutr. (2022) 73:726–37. doi: 10.1080/09637486.2022.2050996 35291893

[52] Xuzhu G Komai-Koma M Leung BP Howe HS McSharry C McInnes IB. Resveratrol modulates murine collagen-induced arthritis by inhibiting Th17 and B-cell function. Ann Rheum Dis. (2012) 71:129–35. doi: 10.1136/ard.2011.149831 21953348

[53] Lu J Zheng Y Yang J Zhang J Cao W Chen X. Resveratrol alleviates inflammatory injury and enhances the apoptosis of fibroblast-like Synoviocytes via mitochondrial dysfunction and Er stress in rats with adjuvant arthritis. Mol Med Rep. (2019) 20:463–72. doi: 10.3892/mmr.2019.10273 31180523 PMC6580038

[54] Farzaei MH Rahimi R Abdollahi M. The role of dietary polyphenols in the Management of Inflammatory Bowel Disease. Curr Pharm Biotechnol. (2015) 16:196–210. doi: 10.2174/1389201016666150118131704 25601607

[55] Niemelä O Aalto M Bloigu A Bloigu R Halkola AS Laatikainen T. Alcohol drinking patterns and laboratory indices of health: does type of alcohol preferred make a difference? Nutrients. (2022) 14:14529. doi: 10.3390/nu14214529 PMC9658819 36364789

[56] Rizzoli R. Dairy products, yogurts, and bone health. Am J Clin Nutr. (2014) 99:1256s–62s. doi: 10.3945/ajcn.113.073056 24695889

[57] Rizzoli R. Dairy products and bone health. Aging Clin Exp Res. (2022) 34:9–24. doi: 10.1007/s40520-021-01970-4 34494238 PMC8794967

[58] de Lamas C de Castro MJ Gil-Campos M Gil Á Couce ML Leis R. Effects of dairy product consumption on height and bone mineral content in children: a systematic review of controlled trials. Adv Nutr. (2019) 10:S88–s96. doi: 10.1093/advances/nmy096 31089738 PMC6518138

[59] Chen W Jiang D Liu K Lyu L Chen Y Sun X. The Association of Milk Products with rheumatoid arthritis: a cross-sectional study from Nhanes. Joint Bone Spine. (2024) 91:105646. doi: 10.1016/j.jbspin.2023.105646 37769799

[60] Frangos T Maret W. Zinc and cadmium in the Aetiology and pathogenesis of osteoarthritis and rheumatoid arthritis. Nutrients. (2020) 13:e53. doi: 10.3390/nu13010053 PMC7824316 33375344

[61] Rondanelli M Faliva MA Tartara A Gasparri C Perna S Infantino V. An update on magnesium and bone health. Biometals. (2021) 34:715–36. doi: 10.1007/s10534-021-00305-0 33959846 PMC8313472

[62] Benito-Garcia E Feskanich D Hu FB Mandl LA Karlson EW. Protein, Iron, and meat consumption and risk for rheumatoid arthritis: a prospective cohort study. Arthritis Res Ther. (2007) 9:R16. doi: 10.1186/ar2123 17288585 PMC1860075

[63] Wang H Zhang R Shen J Jin Y Chang C Hong M. Circulating level of blood Iron and copper associated with inflammation and disease activity of rheumatoid arthritis. Biol Trace Elem Res. (2023) 201:90–7. doi: 10.1007/s12011-022-03148-z 35344152 PMC9823016

[64] Ali ET Jabbar AS Mohammed AN. A comparative study of interleukin 6, inflammatory markers, ferritin, and hematological profile in rheumatoid arthritis patients with anemia of chronic disease and iron deficiency anemia. Anemia. (2019) 2019:3457347. doi: 10.1155/2019/3457347 31057960 PMC6463678

